# Conversion of a soluble protein into a potent chaperone *in vivo*

**DOI:** 10.1038/s41598-019-39158-6

**Published:** 2019-02-25

**Authors:** Soon Bin Kwon, Kisun Ryu, Ahyun Son, Hotcherl Jeong, Keo-Heun Lim, Kyun-Hwan Kim, Baik L. Seong, Seong Il Choi

**Affiliations:** 1 Department of Biotechnology, College of Life Science and Biotechnology, Yonsei University, Seoul, 03722 Republic of Korea; 2 Department of Pharmacy, Ewha Womans University, Seoul, 03760 Republic of Korea; 3 Department of Pharmacology, Center for Cancer Research and Diagnostic Medicine, IBST, School of Medicine, Konkuk University, Seoul, 05029 Republic of Korea; 4 Vaccine Translational Research Center (VTRC), Yonsei University, Seoul, 03722 Republic of Korea; 5 Present Address: Department of Biochemistry and Biophysics, Stockholm University, SE-106 91 Stockholm, Sweden

**Keywords:** Chaperones, Chaperones, Protein aggregation, Protein aggregation

## Abstract

Molecular chaperones play an important role in cellular protein-folding assistance and aggregation inhibition. As a different but complementary model, we previously proposed that, in general, soluble cellular macromolecules with large excluded volume and surface charges exhibit intrinsic chaperone activity to prevent aggregation of their connected polypeptides irrespective of the connection type, thereby contributing to efficient protein folding. As a proof of concept, we here demonstrated that a model recombinant protein with a specific sequence-binding domain robustly exerted chaperone activity toward various proteins harbouring a short recognition tag of 7 residues in *Escherichia coli*. The chaperone activity of this protein was comparable to that of representative *E. coli* chaperones *in vivo*. Furthermore, *in vitro* refolding experiments confirmed the *in vivo* results. Our findings reveal that a soluble protein exhibits the intrinsic chaperone activity to prevent off-pathway aggregation of its interacting proteins, leading to more productive folding while allowing them to fold according to their intrinsic folding pathways. This study gives new insights into the plausible chaperoning role of soluble cellular macromolecules in terms of aggregation inhibition and indirect folding assistance.

## Introduction

How proteins fold efficiently and maintain their solubility in the crowded cellular environment has been a fundamental yet unsolved question^1–4^. Protein aggregation is closely associated with numerous human disorders including neurodegeneration^5^. Molecular chaperones and principles underlying their action mechanisms have provided conceptual frameworks for understanding protein-folding assistance, aggregation inhibition, and proteostasis *in vivo*^6,7^. Chaperones generally assist protein folding by preventing off-pathway “intermolecular” aggregation, often at the expense of the “intramolecular” folding rate or thermodynamic stability of substrate proteins^8–10^. In contrast, in some cases, chaperones can increase the folding rate of client proteins as folding catalysts^11–13^. Of note, protein aggregation can be controlled independently of (or with sacrificing) intramolecular folding and stability^4^. For example, protein in the cavity of GroEL/GroES (GroELS) is safe from aggregation regardless of its conformation, folding, and stability. Consistently, although crucial for protein aggregation inhibition (or protein solubility) by desolvation penalty/intermolecular repulsions, surface charged residues of proteins can be dispensable for protein folding and stability^14^. These observations mean that final productive folding yield is determined by two different routes, intramolecular folding and intermolecular aggregation. Such a distinction not only allows both effects (folding rate increase and aggregation inhibition with folding rate decrease) of chaperones to reconcile without a conceptual conflict, but also highlights the importance of forces of chaperones, as well as other cellular macromolecules, that prevent their connected polypeptides from aggregation^4^.

Chaperones generally recognize and bind to the exposed hydrophobic residues of non-native polypeptides, thereby enabling protein quality control^3,6,7^. These findings led to a widely accepted principle of hydrophobic interaction-mediated substrate recognition and stabilization against aggregation. However, the following non-hydrophobic interaction-driven substrate recognition of chaperones and protein aggregation appear to be difficult to rationalize based solely on direct hydrophobic interaction-driven aggregation inhibition. Chaperones, such as Spy and Trigger Factor (TF), can recognize and bind to the surface-exposed charged regions of substrates^15,16^. The endoplasmic reticulum lectin chaperones calnexin and calreticulin bind to the carbohydrate parts of their substrate proteins^17^. Moreover, GroEL and TRiC/CCT can recognize their substrates mainly through electrostatic interactions^18,19^. Notably, a nucleoplasmin, first coined as a molecular chaperone, recognizes the folded but aggregation-prone histone monomer by electrostatic interactions, and the aggregation is driven by electrostatic interactions^20^. Hydrogen bonds are important for amyloid fibril formation. Intriguingly, the surface-charge patches of heat-shock protein 90 (HSP90) are crucial for the anti-aggregation activity for its substrate proteins, although they are not involved in the substrate-binding^21^. Similarly, the substrate-stabilizing ability of DnaK, an *E. coli* homolog of HSP70, was reported to result largely from the N-terminal domain rather than the C-terminal substrate-binding domain in the context of covalent fusion^22^. DnaK binds to short linear peptides with 2–4 consecutive hydrophobic residues flanked by a basic residue^23^. Direct hydrophobic interactions between DnaK and its substrate protein are very limited. The electrostatic and steric repulsions of DnaK were suggested to play an important role in substrate stabilization besides the hydrophobic interactions^22^. Contrary to the amenable quantitative characterization of the bimolecular interaction forces between chaperones and proteins, quantitative elucidation of the forces (or factors) of chaperones, as well as other cellular macromolecules, responsible for stabilizing their bound substrates against aggregation, remains a great challenge due to the inherent difficulty of this study.

We previously proposed the *cis*-acting protein-folding helper systems, which appear to operate differently from the classical *trans*-acting chaperones^4,24^. A hallmark feature of the cellular protein-folding environment is that nascent polypeptides are tethered to cellular macromolecules with large excluded volume and surface charges, such as ribosomes (2000–3200 kDa), membranes, or cotranslationally folded domains in multidomain proteins. *De novo* protein folding on these cellular macromolecules has been a major issue in terms of chaperone function^7,25–27^, but the tethering effect of such macromolecules has long been underappreciated. Based on the robust chaperone-like activity of these macromolecules, as well as a variety of highly soluble proteins, toward various heterologous aggregation-prone proteins in the fusion context (or *in cis*)^28–31^, this *cis*-acting chaperone-like type was proposed to play an important role in *de novo* folding of endogenous proteins^24^. Consistent with this *cis*-acting model, several lines of evidence indicate that cytosol-exposed nascent chains tethered to ribosomes are aggregation-resistant and co-translational folding-competent^32–35^. Remarkably, intermolecular repulsive forces, such as steric and electrostatic repulsions by the excluded volume and surface charges of cellular macromolecules, were proposed to stabilize their tethered polypeptides against aggregation while the polypeptides can fold in the linkage context^4,24^. This stabilizing mechanism can underlie the intrinsic chaperone activity of soluble cellular macromolecules. The magnitudes of these intermolecular repulsive forces were suggested to increase corresponding to the size and surface charges of molecules^22,24^. Notably, these two forces have been well known as major factors in stabilizing colloids against aggregation^36,37^. Similarly, entropic bristling and hydration by the excluded volume and charged residues of intrinsically disordered proteins or regions were proposed to solubilize their fused proteins^38,39^. Moreover, the entropic pulling forces of HSP70 resulting from its excluded volume repulsions were proposed to underlie its diverse functions^40^.

Large excluded volume and surface charges are the common intrinsic properties of soluble cellular macromolecules including chaperones. This prompted us to hypothesize that cellular macromolecules exhibit intrinsic chaperone activity^4,41^. To test this hypothesis, we here constructed a *trans*-acting artificial chaperone system. Our results reveal that a model soluble protein exhibits intrinsic chaperone activity, which is manifested, upon binding to aggregation-prone proteins in *E. coli*.

## Results

### Design of an artificial chaperone system for showing the intrinsic chaperone activity of a soluble protein

The aforementioned intrinsic chaperone activity of soluble cellular macromolecules can allow them to maintain their connected polypeptides in an aggregation-resistant and folding-competent state even without direct binding to aggregation-prone regions. For example, megadalton-sized ribosomes with supernegative charges on their surface could act as a *cis*-acting folding helper by simply tethering to the carboxyl termini of newly synthesized polypeptides during *de novo* protein folding. Similarly, this can happen if a soluble protein recognizes and binds to a limited terminal region of its client protein via noncovalent interactions (or *in trans*). Thus, a soluble protein can be converted into a chaperone if there is a mean of physical connection with the flanking terminal region of aggregation-prone proteins *in trans*. To demonstrate this, we here designed an artificial recombinant chaperone system.

**Figure 1:**
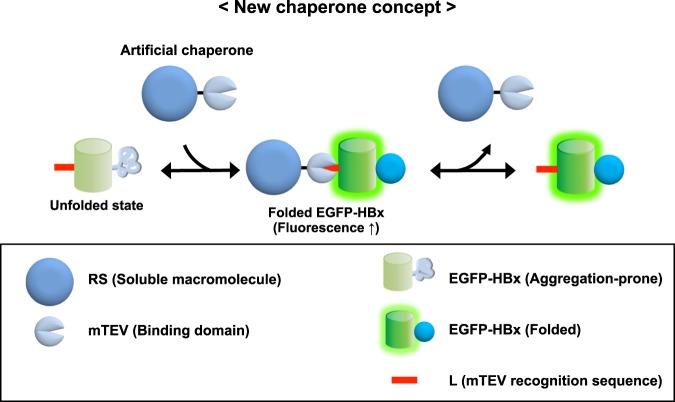
Experimental design for conversion of a soluble protein into a chaperone. Schematic diagram for the construction of an artificial chaperone system to assess the intrinsic chaperone activity of soluble cellular macromolecules. A TEV protease-domain mutant (mTEV) with no proteolytic activity but with a binding ability toward its canonical sequence of 7 residues (denoted as “L”; red bar) was fused to the C-terminus of *E. coli* RS, yielding an artificial chaperone, RS-mTEV. EGFP-HBx harbouring “L” tag is a client protein of RS-mTEV.

A model soluble protein (RS-mTEV) as an artificial chaperone was constructed to specifically bind to a short flanking tag of 7 residues located at the N-terminus of substrate protein (Fig. 1). RS-mTEV consists of two modules. As a tag-binding module of RS-mTEV, we chose a mutant protease domain of tobacco etch virus (mTEV) with no proteolytic activity but still maintaining the binding affinity for its canonical recognition sequence (ENLYFQG)^42^. This protease domain is marginally soluble when expressed alone at 37 °C^43^. To increase mTEV solubility, it was fused to the C-terminus of *E. coli* lysyl tRNA synthetase (RS; 57 kDa), known as a solubility-enhancing fusion partner^31^, yielding a more soluble RS-mTEV protein (see Supplementary Fig. S1). As a client protein of RS-mTEV, enhanced green fluorescent protein (EGFP) was fused to hepatitis B virus X protein (HBx) with intrinsically disordered regions^44^, to yield L-EGFP-HBx where “L” denotes the recognition sequence (ENLYFQG) of mTEV. This model system was designed to minimize direct binding except for the “L” tag between RS-mTEV and its client protein during folding and aggregation in order to assess the intrinsic chaperone activity of RS-mTEV. Moreover, by comparing the chaperone activity between mTEV and RS-mTEV with the same substrate-binding module, we distinguished between the contributions of the two modules (RS and mTEV) to RS-mTEV chaperone activity in the present study.

### RS-mTEV acts as a potent chaperone for its client proteins *in vivo*

**Figure 2:**
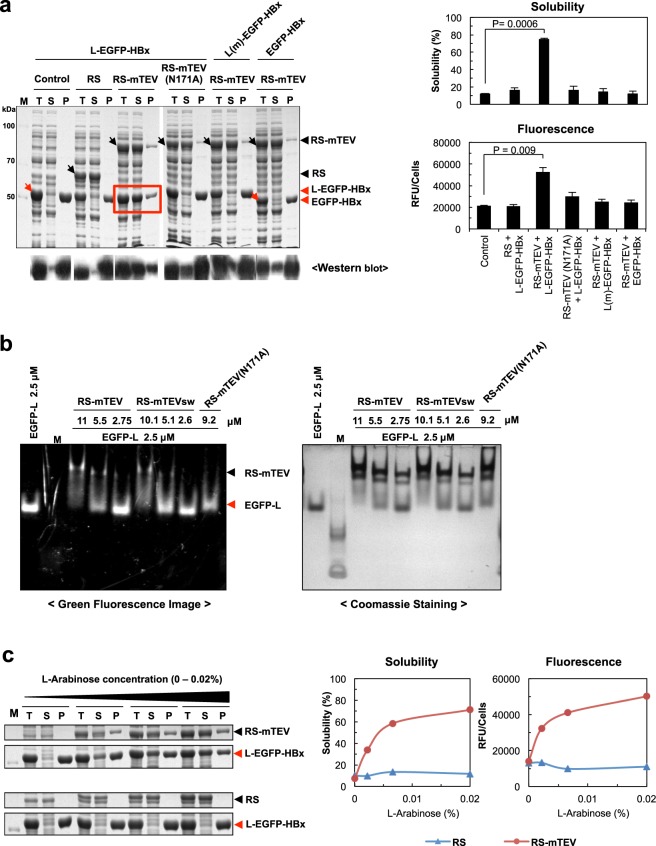
RS-mTEV exhibits potent chaperone activity upon binding to aggregation-prone proteins in *E. coli*. (**a**) Effect of co-expression of RS-mTEV on the solubility and folding of L-EGFP-HBx. RS-mTEV was co-expressed with L-EGFP-HBx, L(m)-EGFP-HBx, and EGFP-HBx, respectively. As negative controls, mock vector pLysE (Control), RS, RS-mTEV(N171A), L(m)-EGFP-HBx, and EGFP-HBx were used. RS-mTEV(N171A) and L(m)-EGFP-HBx harbour mutations in mTEV and “L” tag, respectively, critical to the substrate protein recognition. Proteins were expressed at 25 °C. The total lysate (T), soluble fraction (S), and pellet (P) of each sample with molecular-weight size marker (M) were subjected to SDS-PAGE and western blot. Both solubility, measured using SDS-PAGE and densitometry, and fluorescence intensity of the EGFP fusion proteins in each soluble fraction were compared. The same analytical methods were used for the following Figs 3–5. Black and red arrows indicate artificial chaperones (equivalent or control) and substrate proteins, respectively. (**b**) Interactions of RS-mTEV and its variants with EGFP-L. The interactions between them were analysed using electrophoretic mobility shift assay on native PAGE gel. Fluorescence image was first captured (left) and then stained with Coomassie brilliant blue (right). (**c**) RS-mTEV concentration-dependent chaperone activity. RS-mTEV co-expression was controlled by different concentrations (0–0.02%) of L-arabinose. RS was used as a control.

We investigated the effect of RS-mTEV co-expression on both L-EGFP-HBx solubility and folding in *E. coli* using two co-expression vectors. Information about these vectors is described in more detail (see Supplementary Fig. S2). RS-mTEV co-expression markedly increased L-EGFP-HBx solubility by ~75%, whereas RS co-expression did not increase the solubility (~16%) similar to the corresponding solubility (~12%) in background cells containing a mock vector pLysE as a control (Fig. 2a). We further confirmed that a specific binding of RS-mTEV to the “L” tag in L-EGFP-HBx increased the protein solubility. Residue N171 in mTEV is important for substrate recognition^42^; therefore, this mutation in RS-mTEV [named RS-mTEV(N171A)] resulted in a significantly impaired substrate-binding ability, as shown below (Fig. 2b). Correspondingly, RS-mTEV(N171A) had no detectable solubility-enhancing ability for the substrate proteins (Fig. 2a). Similarly, the solubility of L(m)-EGFP-HBx with a mutation in the “L” tag (ENLYFQG to YNLEFQG) did not respond to RS-mTEV co-expression. Other mutations in the conserved recognition sequence of “L” tag consistently resulted in little or no effect of RS-mTEV coexpression on the solubility of proteins such as L(m)-EGFP-HBx (see Supplementary Fig. S3). As expected, EGFP-HBx solubility without the recognition sequence “L” was unaffected by RS-mTEV co-expression (Fig. 2a). These results clearly demonstrated that RS-mTEV increased protein solubility via its specific binding to the “L” tag in L-EGFP-HBx *in vivo*. Western blot analysis of the substrate proteins using an anti-GFP antibody was in accordance with their corresponding expression patterns on the above sodium dodecyl sulphate polyacrylamide gel electrophoresis (SDS-PAGE) results. We then investigated the folding quality of the proteins solubilized by RS-mTEV co-expression by measuring EGFP fluorescence intensity in soluble fractions containing the client proteins. Solubility enhancement or aggregation inhibition, as important elements for biological relevance, does not necessarily represent proper folding. We observed correlations between EGFP fluorescence intensity and L-EGFP-HBx solubility resulting from RS-mTEV co-expression (Fig. 2a), indicating that RS-mTEV increased both solubility and productive folding yield of its client protein. These results support that RS-mTEV exhibits the intrinsic chaperone activity.

To support the *in vivo* results, we investigated the interaction of RS-mTEV, RS-mTEVsw (a more soluble version of RS-mTEV, which is described in Fig. 5), and RS-mTEV(N171A) with EGFP-L using electrophoretic mobility-shift assay (Fig. 2b). Protein mixtures separated on native-PAGE gel were visualized by detecting the fluorescence of unbound and bound EGFP-L. The amounts of bound EGFP-L, which shifted relatively upward on the gel, increased similarly in cases of RS-mTEV and RS-mTEVsw with raising their concentration. The smearing of the bound forms on the gel suggests that they might dissociate during electrophoresis. The mTEV has been reported to bind to a short peptide containing “L” tag with a dissociation constant K_d_ of 47 μM, measured by differential scanning calorimetry^45^. Compared to RS-mTEV, the amounts of bound EGFP-L in case of RS-mTEV(N171A) substantially decreased, consistent with their *in vivo* effects.

We investigated the dosage effects of co-expressed RS-mTEV on L-EGFP-HBx solubility and folding. The increased amounts of co-expressed RS-mTEV protein with increasing L-arabinose concentration (0%, 0.0022%, 0.0066%, and 0.02%) promoted both L-EGFP-HBx solubility and EGFP fluorescence in an RS-mTEV dosage-dependent manner (Fig. 2c). In contrast, the increase in RS co-expression level had no effect on L-EGFP-HBx solubility and EGFP fluorescence under the same condition (Fig. 2c). However, this RS-mTEV dosage-dependent experiments using arabinose promoter need to be interpreted carefully, although the difference in chaperone activity between the presence and absence of arabinose are obvious. The arabinose promoter-controlled genes have been reported to be expressed in all-or-none fashion at suboptimal concentrations of arabinose; arabinose increases the subpopulation of fully induced cells instead of the whole population with arabinose concentration-dependent expression^46^.

### RS-mTEV acts as a chaperone independently of the recognition-tag position

**Figure 3:**
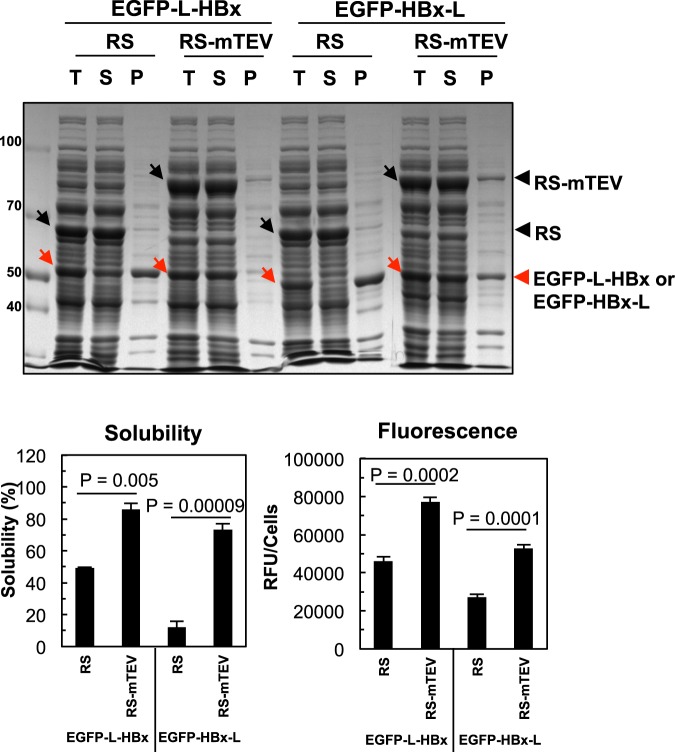
RS-mTEV acts as a chaperone, independently of the recognition tag-position. The “L” tag was placed either in the linker region between EGFP and HBx (EGFP-L-HBx) or at the C-terminus of protein (EGFP-HBx-L). Effect of RS-mTEV coexpression on the solubility and fluorescent intensity of EGFP-L-HBx and EGFP-HBx-L was analysed as described in Fig. 2. RS was used as a control.

One of the advantages of our artificial chaperone system is that the position of the recognition tag in substrate proteins can be changed. The intrinsic chaperone activity of RS-mTEV led us to predict that RS-mTEV should act as a chaperone, independently of the position of the recognition tag in the substrate proteins. To test this scenario, the “L” tag was placed either in the middle between EGFP and HBx (EGFP-L-HBx) or at the C-terminus of the protein (EGFP-HBx-L). RS-mTEV co-expression increased the solubility of both EGFP-L-HBx and EGFP-HBx-L (from 49% to 86% and from 12% to 73%, respectively) (Fig. 3). Consistently, the EGFP fluorescence intensity of the substrate proteins in the soluble fractions was positively correlated with their solubility (Fig. 3). These results showed that RS-mTEV acted as a chaperone for its substrate proteins independently of the recognition-tag position, giving further credence to the intrinsic chaperone activity of RS-mTEV. In the cases of L-EGFP-HBx and EGFP-L-HBx, we do not know whether RS-mTEV acted co- or post-translationally. The case of EGFP-HBx-L clearly indicated that RS-mTEV acted at least post-translationally, thereby broadening the generality of our system.

### RS-mTEV is comparable to classical chaperones

**Figure 4:**
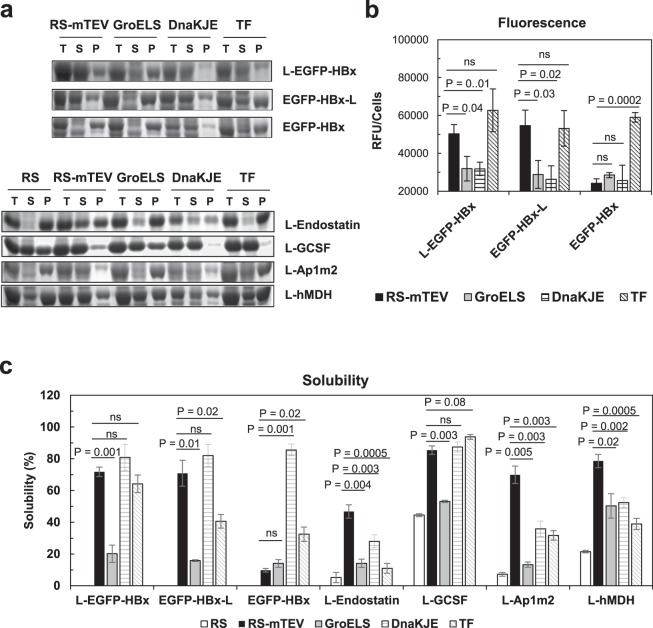
Comparison of RS-mTEV with the representative *E. coli* chaperones. (**a**) Client proteins co-expressed with RS, RS-mTEV, GroELS, DnaKJE, and TF, respectively, were L-EGFP-HBx, EGFP-HBx-L and EGFP-HBx, as well as endostatin, GCSF, Ap1m2, and hMDH. The SDS-PAGE patterns of the expressed proteins were highlighted. (**b**) Comparison of EGFP fluorescence of L-EGFP-HBx, EGFP-HBx-L, and EGFP-HBx of the results shown in (**a**). (**c**) Comparison of protein solubility of the substrate proteins in (**a**).

The representative chaperones, including GroELS, DnaK-DnaJ-GrpE system (DnaKJE), and TF, have been well known to prevent aggregation and assist protein folding in *E. coli*^7,8,27^. Here, we compared RS-mTEV chaperone activity with those of these classical chaperones for L-EGFP-HBx, EGFP-HBx-L, and EGFP-HBx proteins. When co-expressed individually with RS-mTEV, GroELS, DnaKJE, and TF, the corresponding solubility of the three substrate proteins were observed to be 72%, 20%, 81%, and 64% for L-EGFP-HBx, 71%, 16%, 82%, and 41% for EGFP-HBx-L, and 9.8%, 14%, 86%, and 33% for EGFP-HBx (Fig. 4a,c). The co-expression of the chaperones was confirmed (see Supplementary Fig. S4). The results showed that, similar to RS-mTEV, DnaKJE and TF substantially increased the solubility of L-EGFP-HBx and EGFP-HBx-L, whereas GroELS increased it slightly. Furthermore, the EGFP fluorescence intensities of the soluble extracts were correlated with the corresponding solubility of the target proteins upon co-expression with each chaperone, except for DnaKJE (Fig. 4b). TF increased both the solubility and folding of client proteins more efficiently than GroELS and DnaKJE. Notably, RS-mTEV was shown to be comparable to TF.

We additionally compared the solubility-enhancing effects of RS-mTEV with the above chaperones for different substrate proteins, including human endostatin, granulocyte colony stimulating factor (GCSF), AP-1 complex subunit mu-2 (Ap1m2), and malate dehydrogenase (hMDH) with the “L” tag at their N-termini. These aggregation-prone proteins have been known to be involved in cell proliferation or signaling pathways^47–50^. Upon individual co-expression of RS, RS-mTEV, GroEL/ES, DnaKJE, and TF, the corresponding solubilities were 5%, 47%, 14%, 28%, and 11% for endostatin; 44%, 85%, 53%, 87%, and 94% for GCSF; 7%, 70%, 13%, 36%, and 32% for Ap1m2; and 22%, 79%, 50%, 52%, and 39% for hMDH, respectively (Fig. 4a,c). These results showed that RS-mTEV robustly increased the solubility of all tested proteins containing the “L” tag, supporting the intrinsic chaperone activity of RS-mTEV.

### RS-mTEV chaperone activity largely results from RS rather than mTEV

**Figure 5:**
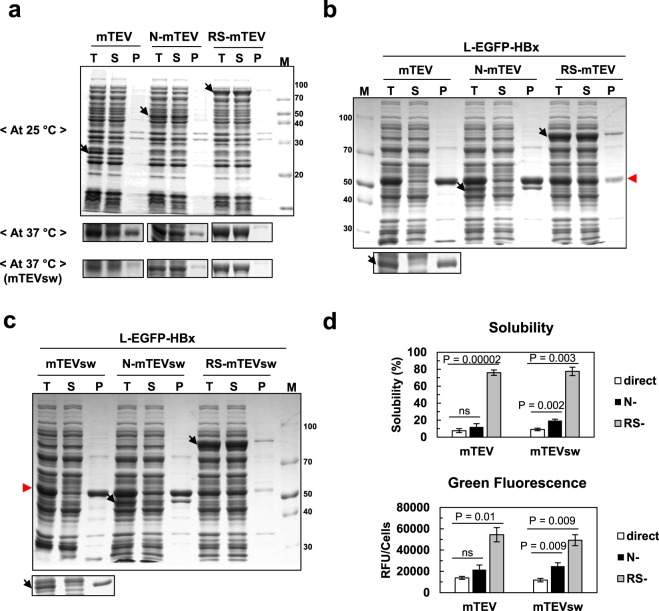
RS-mTEV chaperone activity is largely dependent on RS rather than mTEV. To distinguish between the contributions of RS and mTEV to the RS-mTEV chaperone activity, the chaperone activities of three proteins (mTEV, N-mTEV, and RS-mTEV) and their corresponding more soluble variants (mTEVsw N-mTEVsw, and RS-mTEVsw) were compared. Here, N represents the N-terminal domain of RS. (**a**) Solubility of mTEV, N-mTEV, RS-mTEV, mTEVsw, N-mTEVsw, and RS-mTEVsw at 25 °C and 37 °C. (**b**) Comparison of the chaperone activities of mTEV, N-mTEV, and RS-mTEV for L-EGFP-HBx at 25 °C. mTEV and its fusion variants are indicated by black arrows, and the red arrow indicates L-EGFP-HBx. (**c**) Comparison of the chaperone activity of mTEVsw N-mTEVsw, and RS-mTEVsw at 25 °C under the same conditions as described in **b**. Highlighted bands below main SDS-PAGE data in (**b,c**) represent mTEV and mTEVsw, respectively. (**d**) Solubility and fluorescence intensity of each sample in (**b,c**).

As described in the Introduction section, the substrate-stabilization against aggregation by the surface charges of HSP90, the N-terminal domain of HSP70, and the intermolecular repulsive (or destabilizing) forces of soluble macromolecules appear to act allosterically; long-range chaperone effects can exist even in the absence of direct contact with aggregation-prone regions of connected polypeptides. This (apparent) allosteric mechanism underlies the concept of intrinsic chaperone activity of soluble cellular macromolecules. One would therefore expect that RS-mTEV chaperone activity might be mediated by RS after RS-mTEV’s binding to the “L” tag of client proteins. To test this, we investigated and compared the chaperone effects of three proteins (mTEV without fusion, N-mTEV [N: N-terminal domain (15 kDa) of RS], and RS-mTEV) on L-EGFP-HBx solubility and folding at a low-temperature (25 °C), where the solubility of all three proteins is high (Fig. 5a). Although three proteins share the same substrate-binding module (mTEV), RS-mTEV was superior to mTEV and N-mTEV at promoting L-EGFP-HBx solubility, whereas N-mTEV chaperone activity was slightly higher than that of mTEV (Fig. 5b,d). To further confirm RS-mediated chaperone activity, we used a more soluble TEV variant (TEVsw)^51^ harbouring the same mutation in the TEV domain to block protease activity, yielding mTEVsw. The mTEVsw without fusion was highly soluble, even at 37 °C (Fig. 5a; see Supplementary Fig. S1). Despite the increased solubility of mTEVsw relative to mTEV, mTEVsw without the RS fusion failed to show detectable chaperone activity for L-EGFP-HBx, whereas RS-mTEVsw consistently increased L-EGFP-HBx solubility (Fig. 5c,d). Significant amounts of both mTEV and mTEVsw were observed to co-precipitate with L-EGFP-HBx (Fig. 5b,c). Consistent with the aforementioned allosteric mechanisms, our findings indicate that RS-mTEV chaperone activity appears to largely result from RS, although mTEV as an “L” tag-binding module is indispensible for the implementation of RS-mTEV chaperone activity. They suggest that mTEV module is not sufficient for describing the aggregation inhibition by RS-mTEV and the RS-mediated allosteric effect.

### *In vitro* refolding experiments support RS-mTEV chaperone function

**Figure 6:**
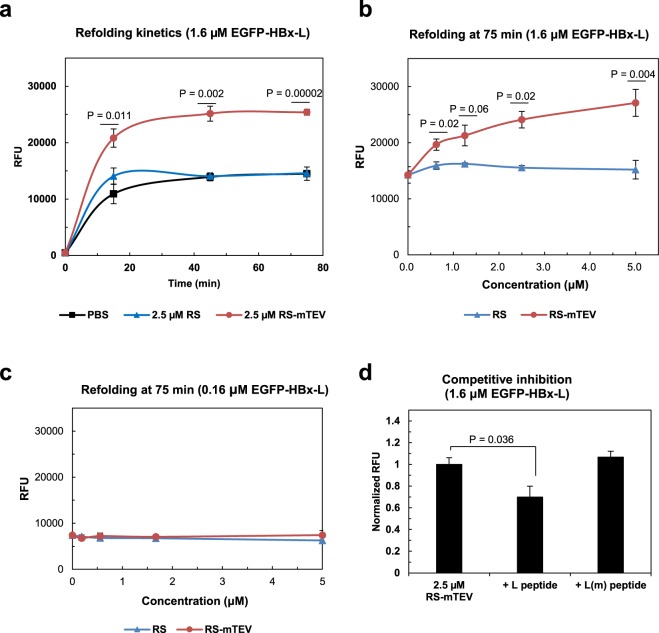
Characterization of the chaperone function of RS-mTEV *in vitro*. (**a**) Refolding kinetics of EGFP-HBx-L (1.6 μM) in the presence of RS-mTEV (2.5 μM) was monitored as a function of time (0, 15, 45, and 75 min). RS and PBS buffer were used as controls. (**b**) Dose-dependent effects of RS-mTEV on EGFP-HBx-L refolding. EGFP fluorescence of the refolded proteins at a concentration (0–5 μM) of RS-mTEV (or RS) was measured at 75 min after initiation of refolding. (**c**) Loss of RS-mTEV chaperone activity at 10-fold lower substrate concentrations as compared with those in (**a**). (**d**) Specific inhibitory effect of the peptide on RS-mTEV chaperone activity. Refolding experiments were similar to those described in **b**, except for the presence of competing (L) or non-competing [L(m)] peptide.

The robust chaperone activity of RS-mTEV *in vivo* occurs in the presence of endogenous chaperones and other folding helper systems. Thus, we cannot exclude the possibility that RS-mTEV simply acts like small HSPs (conventionally called holdase) to prevent aggregation and that it has to collaborate with endogenous folding assistance chaperones for proper protein folding. To characterize RS-mTEV chaperone function more clearly, *in vitro* refolding experiments using EGFP-HBx-L as a substrate protein were performed in the presence and absence of RS-mTEV. GuHCl-denatured EGFP-HBx-L (80 μM) was 50-fold diluted into the refolding buffer containing 2.5 μM RS-mTEV, RS, or phosphate-buffered saline (PBS), and refolding was monitored by following EGFP fluorescence at various time points (0–75 min) at 30 °C. RS-mTEV increased the final refolding yield by ~1. 7 fold, compared to RS and PBS (Fig. 6a). The refolding yields were increased with an RS-mTEV concentration dependence (Fig. 6b). In contrast, RS failed to show any detectable chaperone activity, even at the highest concentration tested (Fig. 6a,b). The final refolding yields of EGFP-HBx-L in the presence of RS-mTEV, RS, and PBS did not converge to the same value (Fig. 6a), indicating that the difference in the final fluorescence signals resulted from irreversible aggregation of the substrate proteins. This implies that RS-mTEV likely assisted protein folding by preventing off-pathway aggregation. To confirm this possibility, *in vitro* refolding was performed at substrate concentrations 10-fold lowered (Fig. 6c), where intermolecular aggregation was minimized. Under these conditions, RS-mTEV chaperone activity was almost similar to the control level (Fig. 6c), indicating that RS-mTEV indirectly assisted protein folding by preventing off-pathway intermolecular aggregation rather than increasing the intramolecular folding rate.

To further confirm *in vitro* RS-mTEV chaperone activity via the specific binding to its canonical recognition sequence in the substrate proteins, we investigated the effect of competing peptides on RS-mTEV chaperone activity. The sequence of the competitive-inhibitor peptide was flanked by TT and GT (TT-ENLYFQS-GT), whereas that of the control peptide represented the inverted form of the canonical recognition sequence (TT-SQFTLNE-GT) harbouring a single point mutation in the middle. Addition of the competitive-inhibitor peptide to the refolding buffer abolished RS-mTEV chaperone activity to the level of the control, whereas the control peptide had no inhibitory effect on RS-mTEV chaperone activity (Fig. 6d). These results demonstrated that *in vitro* RS-mTEV chaperone activity resulted from its specific binding to the canonical recognition sequence. In accordance with the *in vivo* results, the overall *in vitro* refolding results showed that RS-mTEV exhibits chaperone activity to prevent aggregation and thus to assist protein folding indirectly, although the simplified artificial *in vitro* conditions in our study are greatly different from *in vivo* environments.

## Discussion

In this study, we have shown that a soluble protein exhibits intrinsic chaperone activity in terms of aggregation inhibition and indirect folding assistance. A soluble model protein, RS-mTEV, displayed a robust chaperone activity for its client proteins by specifically binding to the “L” tag of 7 residues (Figs 2, 3 and 4). The fluorescence intensity of EGFP-HBx fusion proteins was followed to assess proper folding because of the absence of an *in vitro* HBx assay^52,53^. In particular, our artificial chaperone system is suitable for exploring the intrinsic chaperone activity of a soluble protein because of the following reasons. The “L” tag is very short and is located at the flanking regions of the client proteins, minimizing the direct interactions between RS-mTEV and client proteins, except for the “L” tag. Moreover, RS-mTEV exhibited a high degree of specificity for the “L” tag (Figs 2a and 6d), consistent with a previous report^42^. Similar to the separation of the client protein into two parts (“L” tag and EGFP-HBx), RS-mTEV comprises two distinct modules, a solubility-enhancing module (RS) and a client-binding module (mTEV), allowing us to distinguish between the contributions of RS and mTEV to the chaperone activity of RS-mTEV (Fig. 5). The RS-mediated allosteric chaperone activity in RS-mTEV is in a good accordance with the chaperone activity mediated by the surface charges of HSP90^21^ and the N-terminal domain of HSP70^22^. All our findings consistently indicate that RS-mTEV exhibits intrinsic chaperone activity, which is manifested, upon binding to aggregation-prone proteins. Of note, using the conventional natural systems in which cellular macromolecules interact directly with the structural regions of target proteins, it is challenging to assess the intrinsic chaperone activity of them. To overcome this difficulty, we designed an artificial recombinant system.

Our overall results show that it is an effective way of achieving productive folding of aggregation-prone proteins to prevent off-pathway aggregation (or increase solubility) through the physical connection with a soluble protein *in trans*. In particular, absence of chaperoning effect of RS-mTEV on the folding of its client proteins in the absence of aggregation *in vitro* (Fig. 6c) strongly indicates the aggregation inhibition-based indirect folding assistance. In a good accordance with the binding mode of RS-mTEV via the flanking “L” tag, RS-mTEV did not interfere the folding of the client proteins (Fig. 6c), indicating that they fold according to their intrinsic folding pathways. A similar action mechanism was proposed for the *cis*-acting folding helpers^4,24^. In the physical linkage context, the surface charges and excluded volume of highly soluble cellular macromolecules were proposed to play an important role in keeping their connected polypeptides in an aggregation-resistant state, thus leading to more productive folding^4,24^. This mechanism *in cis* appears to well explain the observed RS-mTEV’s behaviours *in trans*, including the “L” tag-mediated substrate binding and aggregation inhibition (Fig. 2), this tag position and client protein type-independent action (Figs 3 and 4), the allosteric effect of RS in RS-mTEV (Fig. 5), and the indirect folding assistance (Fig. 6), although there is no evidence for the aggregation inhibition by the surface charges and excluded volume of RS-mTEV. The intrinsic chaperone activity of a soluble protein presented here suggests a possibility that soluble cellular macromolecules have the potential to exert beneficial effect on the folding and solubility of aggregation-prone polypeptides including their non-native state as well as native state, provided there is a mean of physical connection between them. However, mTEV and mTEVsw alone did not increase the solubility of client proteins and instead they co-precipitated together (Fig. 5b,c). Because the original mTEV is marginally soluble (Fig. S1), it is likely that the soluble mTEV and mTEVsw might not have the capacity to maintain highly aggregation-prone client proteins in a soluble state. RS, non-substrate binding module, in RS-mTEV likely increases such capacity of soluble mTEV, consistent with the previous findings that non-substrate binding (or non-direct linking) modules significantly affect the aggregation of indirectly connected proteins^4,21,22,24^.

RS-mTEV’s interaction with the client proteins through the flanking “L” tag indicate that it binds to native state (Fig. 2b) as well as non-native state as shown in the coprecipitation of mTEV with the client proteins (Fig. 5b). Such binding of RS-mTEV to the folded state raises a question if our definition of RS-mTEV as a chaperone in terms of aggregation inhibition and indirect folding assistance is valid. By definition, molecular chaperones assist folding of client proteins through transient interactions; they are not components of the final native structures^54^. This definition led to a general belief that chaperones do not bind to the final native structures. However, the following findings support the notion that chaperones “preferentially” bind to non-native state relative to folded state. TF, Spy, calnexin/calreticulin, and DnaK have been known to exhibit a significant binding affinity for native-like or native structures^55–58^. For example, Spy binds to the unfolded-like, intermediate-like, and folded conformers of Im7, its client, with K_d_ of 10.4 μM, 3.5 μM and 20.5 μM, respectively^55^. DnaK binds to native σ^32^ with K_d_ of 1.4 μM^58^. The chaperone concept has been extended to the artificial fusion proteins. Solubility-enhancing fusion partners (or solubility enhancers), such as thioredoxin and maltose-binding protein, were named as molecular chaperones in the fusion context for promoting solubility and proper folding of their attached proteins^43,59^. Thus, the binding of RS-mTEV to the folded clients via the flanking “L” tag (Fig. 2b) can be compatible with the current broad chaperone concept. RS-mTEV increased the productive folding yield by preventing aggregation (Figs 2 and 6). In this regard, RS-mTEV is similar to folding chaperones but different from sHSPs as holdases that stop the substrate folding in the complexed state. Seemingly, the connection type *in trans* between RS-mTEV and its client through the short flanking “L” tag (Fig. 1) is similar to that *in cis* between cellular macromolecules (ribosomes, membrane surfaces, or folded domains) and their tethered nascent chains. RS-mTEV chaperone type, although artificial, might be physiologically relevant to *de novo* protein folding *in vivo*.

The concept of the intrinsic chaperone activity of a soluble protein based on aggregation inhibition and indirect folding assistance might be applicable to soluble cellular macromolecules because such intrinsic chaperone activity of cellular macromolecules can be effective upon physical connection with aggregation-prone polypeptides. Aggregation-prone polypeptides in the crowded cytosol are physically connected to a variety of cellular macromolecules. Individual proteins are estimated to continuously interact with five putative partners in the *E. coli* cytoplasm^60^, making quinary interactions with macromolecules inside cells^61^. Previously, the *cis*-acting intrinsic chaperone-like activity of soluble proteins and domains were suggested based on *in vivo* results^24^. To more clearly define the intrinsic chaperone activity of cellular macromolecules, we here designed the *trans*-acting artificial chaperone system, allowing the independent control of RS-mTEV *in vivo* and *in vitro*. Interestingly, classical chaperones can be converted into potent *cis*-acting chaperones^22,62,63^, and ribosomes can act as chaperones both *in trans* and *in cis*^28,35,64–66^. Here, RS, a potent solubility enhancer *in cis*^31^, provides the *trans-*acting chaperone activity as a component of RS-mTEV (Fig. 5). Similarly, endoprotease DegP (HtrA) can be converted into a chaperone under different conditions^67^, with many other protease components previously shown to exhibit chaperone-like activity^68^. Moreover, various RNAs, highly soluble macromolecules, have been increasingly reported to act as potent chaperones^31,35,69–73^. The concept of intrinsic chaperone activity of a soluble protein in our study is helpful for understanding the aforementioned diverse chaperone types although we do not show the molecular origin of the intrinsic chaperone activity of RS-mTEV.

The (apparent) allosteric effect of RS in RS-mTEV on its client protein (Fig. 5) might provide new insights into therapeutic intervention in aggregation-associated diseases. Consistent with our findings, a bulky protein conjugated to amyloid-specific binding dye dramatically inhibits the amyloid formation due to the protein’s steric hindrance or excluded volume repulsion^74^. Our findings can rationalize why even cellular macromolecules, which bind to the flanking or remote sites away from aggregation-prone regions in proteins, might be potential drug targets for protein aggregation-associated diseases; for example, small chemicals that modulate the expression level or stability of the cellular macromolecules could be drug candidates. This idea holds promise, considering that the aggregation-prone proteins associated with diseases interact directly or indirectly with a variety of cellular macromolecules.

Taken together, the present study on the intrinsic chaperone activity of RS-mTEV provides new insights into the chaperoning role of cellular macromolecules, which is related to cellular protein folding, aggregation, protein solubility maintenance, aggregation-associated diseases, and recombinant protein production technology. Currently, a variety of short peptides and their specific binding proteins including antibodies are available. Our approach here can be applicable to their specific sets, contributing to the elucidation of the role of the excluded volume and surface properties of cellular macromolecules in the folding and aggregation of their connected polypeptides.

## Methods

### Cloning

We used two different types of vectors for co-expression (pGE and pLysE vectors) (see Supplementary Fig. S2). The pGE vectors originated from pGE-LysRS^31^, and pLysE vectors were obtained from Merck Chemicals GmbH (Novagen; Darmstadt, Germany). First, the pBAD promoter was inserted into the *Nru*I and *Ava*I restriction sites of pLysE, producing the pLysEpBAD vector with a new *Hpa*I site at the flanking regions of the pBAD promoter. We inserted the sequences for RS, RS-mTEV, variants of RS-mTEV, and the molecular chaperones into the pLysEpBAD vector, respectively. Sequences for client proteins, including EGFP-HBx variants, endostatin, GCSF, Ap1m2, and hMDH, were inserted into *Nde*I and restriction sites in the multi-cloning site (*Hind*III, *Hind*III, *Sal*I, and *Hind*III, respectively) in the presence or absence of the TEV protease-recognition sequence in pGE vector. The MSEQ amino acid sequence was inserted at the N-termini of substrate proteins to increase the expression level of proteins (see Supplementary Fig. S5).

### Protein expression

Competent cells [*E. coli* BL21(DE3)] were co-transformed with the aforementioned vectors, and cells were grown as previously reported^31^, with some modifications. The expression of proteins from the pLysEpBAD vector was induced with 0.02% L-arabinose unless otherwise mentioned, followed by culturing at 25 °C for 1.5 h. Then, the cells were treated with IPTG tailored to the expression of each client protein as follows: 50 μM for EGFP-HBx and hMDH, 75 μM for endostatin, 200 μM for GCSF, and 150 μM for Ap1m2. Cells treated with IPTG were cultured at 25 °C for an additional 4 h. The protein solubility was measured following a previous protocol^24^. In brief, the harvested cell pellets obtained from 2.5 mL of culture by brief centrifugation were suspended in 0.3 mL of PBS and sonicated. Twenty microliters of the lysates corresponding the total fraction was sampled. The remaining cell lysates were centrifuged (15000 × g for 12 min) to separate soluble fraction and insoluble fraction. PBS (280 μL) was added to the insoluble fraction and then sonicated briefly for resuspension. The total, soluble, and insoluble fractions (20 μL) were mixed with 20 μL of 2 × SDS buffer. After boiling for 3 min, the cooled samples at room temperature were centrifuged briefly. Each fraction was loaded onto SDS-PAGE to analyse the solubility of each target protein. The loading volume was normalized by the concentration of the harvest cells. Protein solubility was measured using SDS-PAGE and densitometry. All experiments with error bars were performed in triplicate.

### Western blot

Lysate samples were loaded onto polyacrylamide gels and transferred to a polyvinylidene difluoride membrane (ISEQ. 00010; Millipore, Billerica, MA, USA) according to a previously reported protocol^75^. Anti-GFP (632377; Clontech Laboratories, Mountain View, CA, USA) and anti-Penta His (34660; QIAGEN, Hilden, Germany) were used as primary antibodies, and anti-rabbit IgG (A6154; Sigma-Aldrich, St. Louis, MO, USA) and anti-mouse IgG (A4416; Sigma-Aldrich, St. Louis, MO, USA) were used as secondary antibodies.

### Fluorescence assay of EGFP-fused proteins

The fluorescence of EGFP-HBx variants was measured to investigate the proper folding of proteins. Each soluble fraction of lysed samples was normalized to cellular optical density and added to the well of a 96-well plate (30496; SPL Life Sciences, Gyeonggi-do, Korea). The fluorescent intensity of samples was determined at 485 nm (excitation) and 520 nm (emission), with FLUOstar OPTIMA (BMG Labtech, Cary, NC, USA) used to measure the fluorescence of each well.

### *In vitro* refolding

Purified EGFP-HBx-L proteins in denaturing buffer [50 mM Tris-HCl (pH 7.5), 300 mM NaCl, 6 M GuHCl, 1 mM DTT, and 1 mM EDTA] were supplemented with 50 mM DTT for 30 min before use. The denatured and reduced protein mixtures were 50-fold diluted into refolding buffer [50 mM Tris-HCl (pH 7.5), 150 mM NaCl, and 5 mM MgCl_2_] and incubated at 30 °C. Different concentrations of RS or RS-mTEV were added to refolding buffer along with 1 mM DTT. For each time-course refolding experiment, four individual samples were prepared for monitoring the refolding reaction as a function of time (0, 15, 45, and 75 min) where 0 min corresponds to the sample before dilution of denatured proteins into the refolding buffer. After the initiation of the refolding for 0, 30, 60 min, the samples were centrifuged at 15,000 *x g* for 15 min at 30 °C, making the total refolding time 15, 45, and 75 min, respectively. Green fluorescence intensity in the supernatant of each sample was measured. We used RS-mTEVsw for the refolding experiments, which exhibits better stability and solubility than its prototype^51^. When testing 10-fold lower concentrations of EGFP-HBx-L, 1 mg/mL bovine serum albumin was added to the refolding buffer to reduce loss of the substrate protein due to nonspecific adsorption. All refolding experiments were performed in triplicate. Peptides (5 mM; TT-ENLYFQS-GT and TT-SQFTLNE-GT) were dissolved in 100% dimethyl sulfoxide and used to inhibit the chaperone effect of RS-mTEV in *in vitro* refolding assays.

### Electrophoretic mobility shift assay (EMSA)

Purified proteins including RS-mTEV (11, 5.5, 2.75 μM), RS-mTEVsw (10.1, 5.1, 2.6 μM), and RS-mTEV(N171A) (9.2 μM) were individually mixed with the folded form of EGFP-L (2.5 μM). EGFP-L alone was used as a control. The mixtures were incubated at room temperature for 30 min and then separated on native PAGE gel. Before staining with Coomassie brilliant blue (EBP-1011; Elpis Biotech, Daejeon, Korea), a fluorescence image of the gel was captured by detecting the fluorescence of EGFP-L in the bound and unbound forms.

### Statistical analysis

The error bars in each graph represent the mean ± standard deviation of results obtained from triplicate experiments. Statistical significance was analysed using Student’s *t* test. A two-tailed P-value was considered statistically significant at P < 0.1.

## Supplementary information

Conversion of a soluble protein into a potent chaperone in vivo

## Data Availability

All data generated or analysed during this study are included in this published article (and its supplementary information files).
