# Publisher Correction: Conversion of a soluble protein into a potent chaperone *in vivo*

**DOI:** 10.1038/s41598-019-46941-y

**Published:** 2019-09-02

**Authors:** Soon Bin Kwon, Kisun Ryu, Ahyun Son, Hotcherl Jeong, Keo-Heun Lim, Kyun-Hwan Kim, Baik L. Seong, Seong Il Choi

**Affiliations:** 1 Department of Biotechnology, College of Life Science and Biotechnology, Yonsei University, Seoul, 03722 Republic of Korea; 2 Department of Pharmacy, Ewha Womans University, Seoul, 03760 Republic of Korea; 3 Department of Pharmacology, Center for Cancer Research and Diagnostic Medicine, IBST, School of Medicine, Konkuk University, Seoul, 05029 Republic of Korea; 4 Vaccine Translational Research Center (VTRC), Yonsei University, Seoul, 03722 Republic of Korea; 5 Present Address: Department of Biochemistry and Biophysics, Stockholm University, SE-106 91, Stockholm, Sweden

Correction to: *Scientific Reports* 10.1038/s41598-019-39158-7, published online 25 February 2019

In the original version of this Article, the authors Soon Bin Kwon and Seong Il Choi were incorrectly indexed. This error has now been corrected.

