# Supplementary material for: Conversion of a soluble protein into a potent chaperone in vivo
Source: Sci Rep. 2019 Feb 25;9:2735. doi: 10.1038/s41598-019-39158-6 (PMC6389997; doi:10.1038/s41598-019-39158-6)
Supplement: Supplementary file 1 — Conversion of a soluble protein into a potent chaperone in vivo [file 41598_2019_39158_MOESM1_ESM.pdf]

Conversion of a soluble protein into a potent chaperone *in vivo*

Supplementary Information

Soon Bin Kwon[1], Kisun Ryu[1], Ahyun Son[1], Hotcherl Jeong[2], Keo-Heun Lim[3], Kyun-Hwan Kim[3], Baik L. Seong[1, 4,]*, & Seong Il Choi [1, 5,]*

[1] Department of Biotechnology, College of Life Science and Biotechnology, Yonsei University, Seoul 03722, Republic of Korea

[2] Department of Pharmacy, Ewha Womans University, Seoul 03760, Republic of Korea

[3] Department of Pharmacology, Center for Cancer Research and Diagnostic Medicine, IBST, School of Medicine, Konkuk University, Seoul 05029, Republic of Korea

[4] Vaccine Translational Research Center (VTRC), Yonsei University, Seoul 03722, Republic of Korea

[5] (Present Affiliation) Department of Biochemistry and Biophysics, Stockholm University, SE-106 91 Stockholm, Sweden.

* Corresponding authors

Correspondence should be addressed to B.L.S. (blseong@yonsei.ac.kr) or S.I.C. (choisi345@gmail.com)

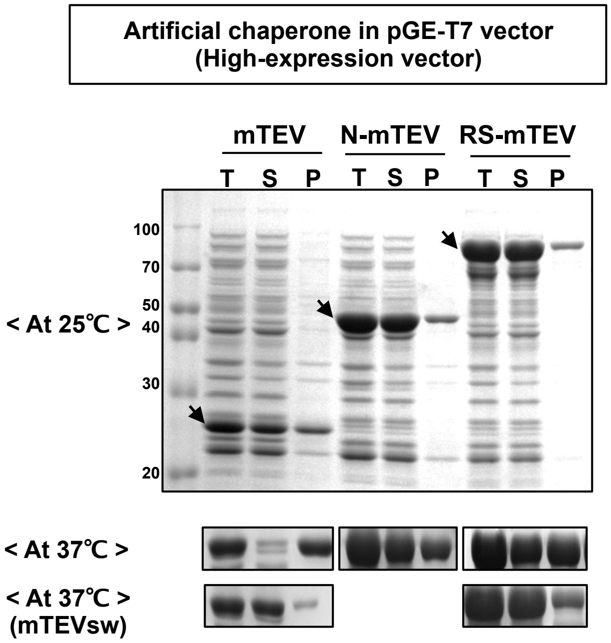

Supplementary Figure S1. Expression of mTEV and its derivatives in *E. coli.* mTEV, N-mTEV, and RS-mTEV were expressed in *E. coli* at 25 °C and 37 °C. N and RS represent the N-terminal domain of *E. coli* LysRS and the whole LysRS, respectively. More soluble mTEV variant (mTEVsw) and its derivative (RS-mTEVsw) were also expressed in *E. coli* at 37 °C. The solubilities of the three mTEV variants expressed at 25 °C were similar, whereas that of mTEV decreased at 37 °C. mTEVsw alone was highly soluble even at 37 °C.

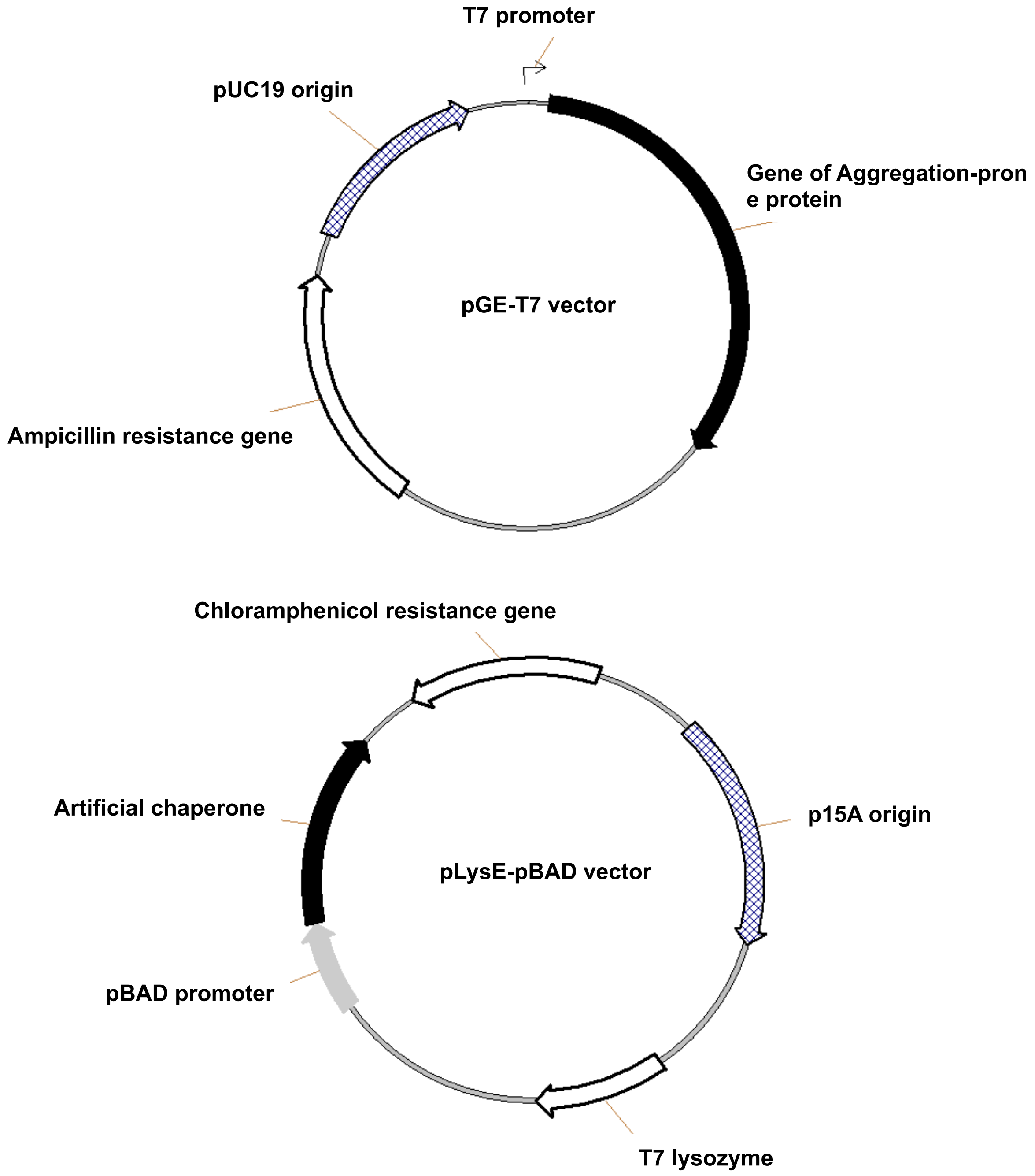

Supplementary Figure S2. Diagram of co-expression vectors used for aggregation-prone proteins and artificial chaperones. pGET7 vector used for the expression of aggregation-prone substrate proteins harbours an ampicillin-resistance gene and a pUC19 origin of replication. Protein expression under control of T7 promoter was induced by IPTG. pLysEpBAD used for chaperone expression carries a chloramphenicol-resistance gene and a p15A origin of replication. Expression of artificial chaperones under the control of the pBAD promoter was triggered by L-arabinose.

L : ENLYFQG
L(m) : YNLEFQG
L(m1) : ENLQFYG
L(m2) : YNLQFEG
L(m3) : QNLEFYG

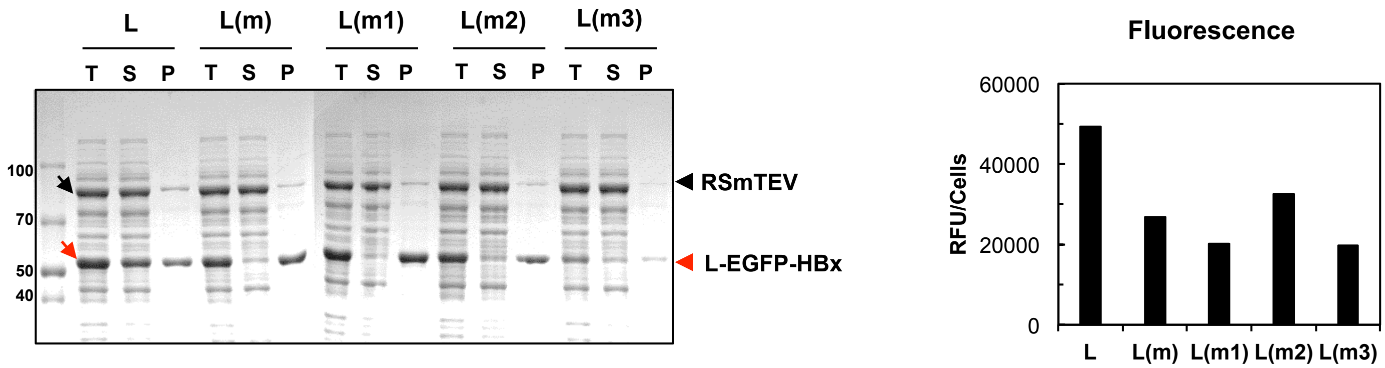

Supplementary Figure S3. **Mutation of the "L" tag in L-EGFP-HBx alters interaction with RS-mTEV.** A mutation was introduced in the conserved residues of the "L" tag, and the resulting mutant variants [L(m), L(m1), L(m2), and L(m3)] were attached to the N-terminus of EGFP-HBx, respectively. These proteins were co-expressed with RS-mTEV in *E. coli*. Mutated sequences in the "L" tag are indicated in red. Expressed proteins were analysed using SDS-PAGE and verified by measuring fluorescence (histogram, right).

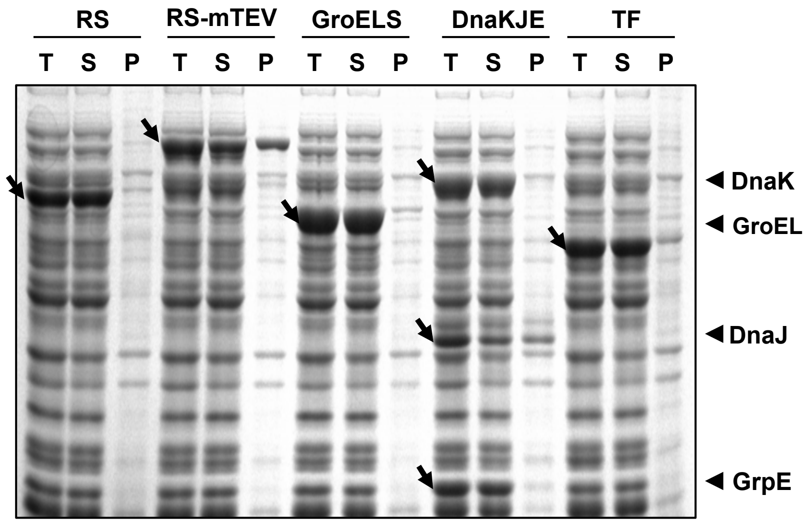

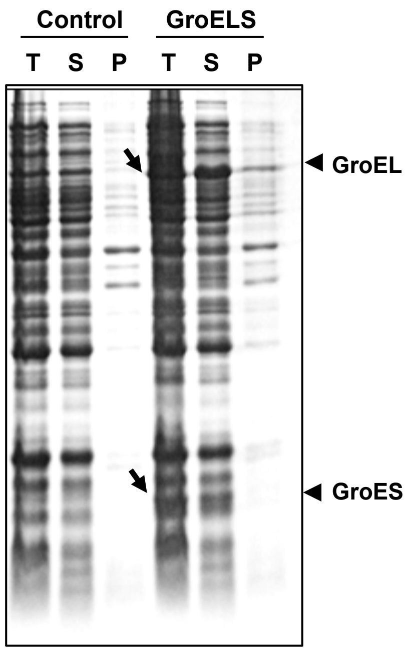

Supplementary Figure S4. Confirmation of the expression of RS, RS-mTEV, and molecular chaperones. RS, RS-mTEV, GroELS, DnaKJE, and TF were expressed in *E. coli*, and their expression was analysed using SDS-PAGE. Each target band is indicated by an arrow. In the case of GroELS, the SDS-PAGE result (down) was added to clearly see the expression of GroES, a relatively small sized protein, which was not shown in the upper SDS-PAGE result obtained after a long running time for a better resolution.

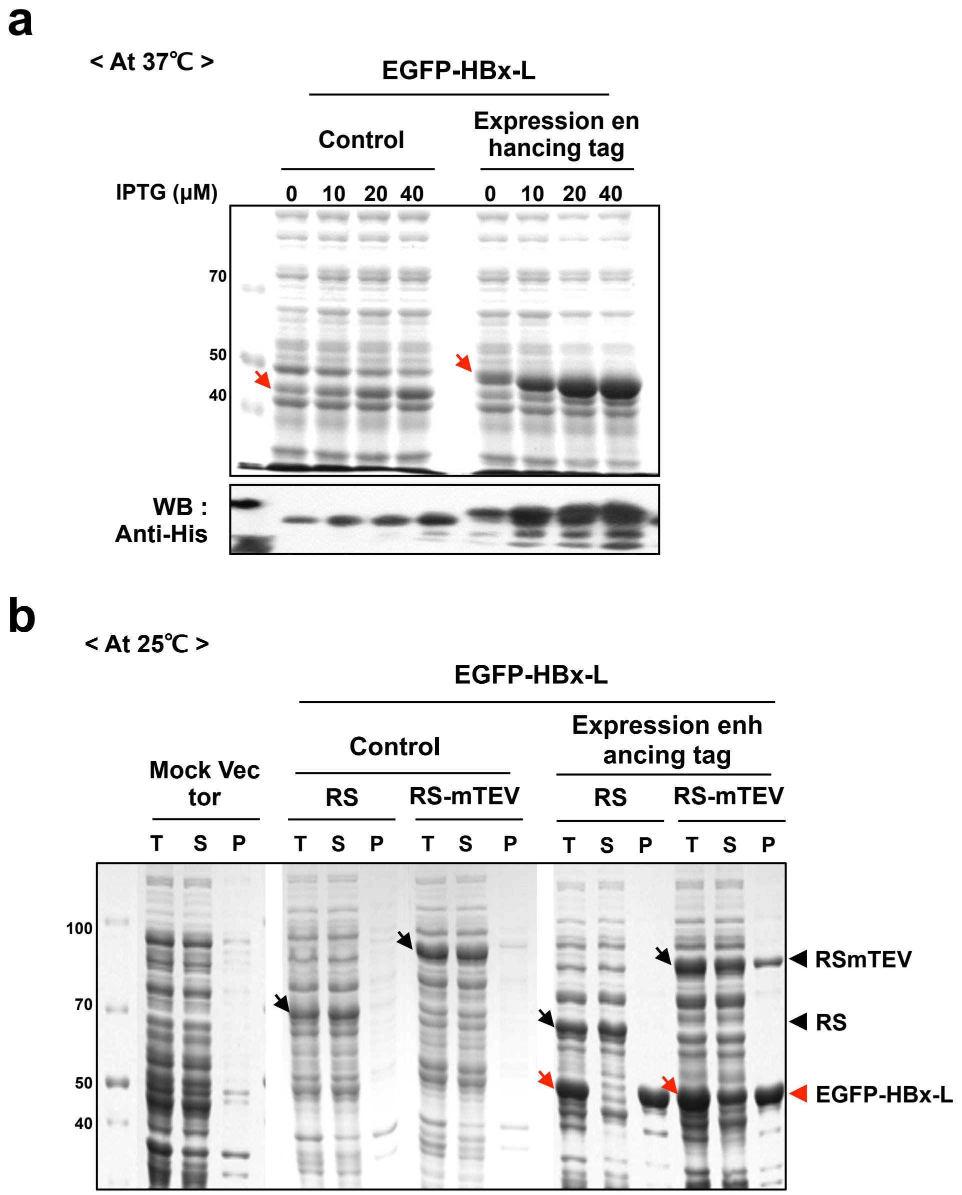

Supplementary Figure S5. The N-terminal MSEQ tag increases the expression of the substrate proteins of RS-mTEV. (**a**) EGFP-HBx-L in the presence or absence of this tag was expressed in *E. coli* at 37 °C and induced at various IPTG concentrations (0–40 μM). Total lysates of each sample were analysed using SDS-PAGE and western blot. Red arrows indicate EGFP-HBx-L expression. (**b**) EGFP-HBx-L in the presence or absence of the tag was expressed in *E. coli* at 25 °C (induced by 100 μM IPTG) along with RS or RS-mTEV co-expression, followed by SDS-PAGE analysis.
